# Valgus and varus deformity after wide-local excision, brachytherapy and external beam irradiation in two children with lower extremity synovial cell sarcoma: case report

**DOI:** 10.1186/1471-2407-4-57

**Published:** 2004-08-27

**Authors:** Daniel T Fletcher, William C Warner, Michael D Neel, Thomas E Merchant

**Affiliations:** 1 Division of Radiation Oncology, Department of Radiological Sciences, St. Jude Children's Research Hospital, 332 N. Lauderdale Street, Memphis, Tennessee, USA; 2 Division of Orthopedics, Department of Surgery, St. Jude Children's Research Hospital, 332 N. Lauderdale Street, Memphis, Tennessee, USA

## Abstract

**Background:**

Limb-salvage is a primary objective in the management of extremity soft-tissue sarcoma in adults and children. Wide-local excision combined with radiation therapy is effective in achieving local tumor control with acceptable morbidity and good functional outcomes for most patients.

**Case Presentation:**

Two cases of deformity after wide-local excision, brachytherapy and external beam irradiation for lower-extremity synovial cell sarcoma are presented and discussed to highlight contributing factors, time course of radiation effects and orthopedic management. In an effort to spare normal tissues from the long-term effects of radiation therapy, more focal irradiation techniques have been applied to patients with musculoskeletal tumors including brachytherapy and conformal radiation therapy. As illustrated in this report, the use of these techniques results in the asymmetric irradiation of growth plates and contributes to the development of valgus or varus deformity and leg-length discrepancies.

**Conclusions:**

Despite good functional outcomes, progressive deformity in both patients required epiphysiodesis more than 3 years after initial management. There is a dearth of information related to the effects of radiation therapy on the musculoskeletal system in children. Because limb-sparing approaches are to be highlighted in the next generation of cooperative group protocols for children with musculoskeletal tumors, documentation of the effects of surgery and radiation therapy will lead to improved decision making in the selection of the best treatment approach and in the follow-up of these patients.

## Background

Limb-salvage is an important treatment objective for adults and children with extremity soft tissue sarcoma and often requires the use of limited surgery and irradiation [1]. Limiting the extent of resection balances the need for radical excision with the need to preserve the functional and structural integrity of the limb and tissues adjacent to those involved with tumor. Radiation therapy has been proven to compensate for incomplete resection or limited resections with involved, close or indeterminate margins as long as the dose and volume are adequate [2-5]. Excellent rates of local control have been achieved for adults and children with extremity soft tissue sarcoma using limb sparing approaches [6-11]. Little is known about the long-term morbidity of the combined effects of limited surgery and irradiation on bone and soft tissue in the pediatric population.

Surgery and radiation therapy both have the potential to cause significant morbidity including loss of function and deformity [6,11-15]. Tumor resection often requires removal of normal tissue compartments and structural elements, even in a limb-sparing approach. This places the patient at risk for complications including destabilization and abnormalities in growth and function. Additive are the effects of high-dose irradiation, which is often required in the treatment of these tumors, and which may compound the effects of resection. The use of chemotherapy, when indicated, may also add to the combined effects of treatment. The timing of surgery and radiation therapy, the operative approach and the selection of the specific radiation treatment modality often depends on a number of important clinical factors including the size and type of tumor, site of involvement, prior surgical manipulation, extent of resection and the potential for as good functional outcome.

Individual cases of valgus and varus deformity after limited surgery and irradiation for extremity soft-tissue sarcoma are presented and discussed to identify factors that may be responsible for these treatment complications. Pre-existing orthopedic problems, multiple attempts at resection, post-operative infection, the use of chemotherapy and the addition of brachytherapy to external beam radiation therapy appear to be contributory. Because limb-sparing approaches will be an important component of the next generation of cooperative group studies for extremity soft tissue sarcoma in children, the incidence and severity of this and other treatment-related complications should be documented as well as efforts to limit the effects of these treatments and identify solutions for established problems.

## Case Presentations

### Case 1

**Figure 1:**
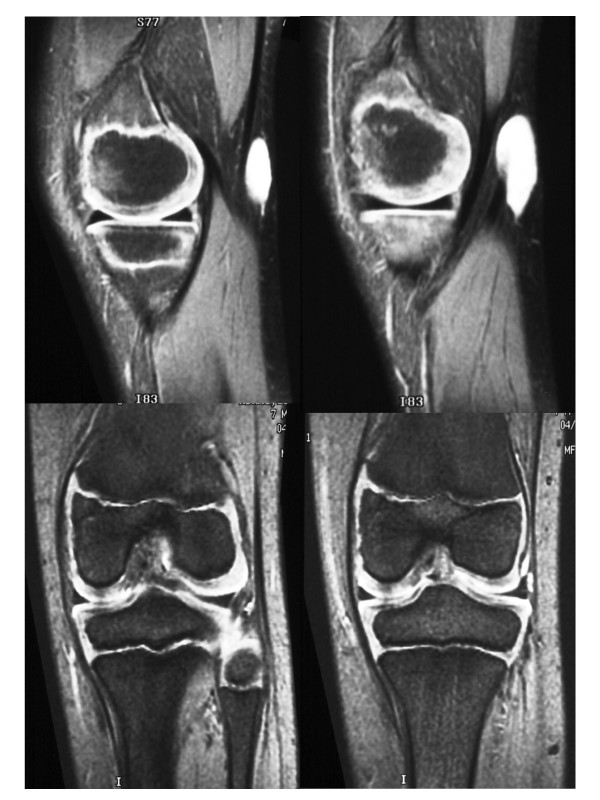
MRI at diagnosis (case one).

At the time of diagnosis, this patient was an 8 year-old male with an approximate 12–24 month history of mild pain and swelling in the left popliteal region. There was no complaint of fever or decreased range of motion. He presented to a local orthopedist (April 1995) and was found to have a palpable abnormality on the posterior aspect of the knee consistent with a Baker's cyst. Aspiration was unsuccessful and the patient was treated with ibuprofen for a 10-day course. Nearly one year later (April 1996) the family sought a second opinion and an MR study was ordered that revealed a cystic structure [Figure 1]. The patient returned to the original orthopedist and was found to have a painful and enlarging mass in the left popliteal region. He underwent resection of a solid and cystic mass (November 1996) measuring 7.0 × 6.0 × 3.0 cm. The tumor was described as a high-grade synovial cell sarcoma. The extent of resection was incomplete with gross residual tumor remaining about the lateral aspect of the knee and external to the joint capsule which also appeared to be the site of origin. The patient was transferred one month later to St. Jude Children's Research Hospital for further evaluation and treatment.

**Figure 2:**
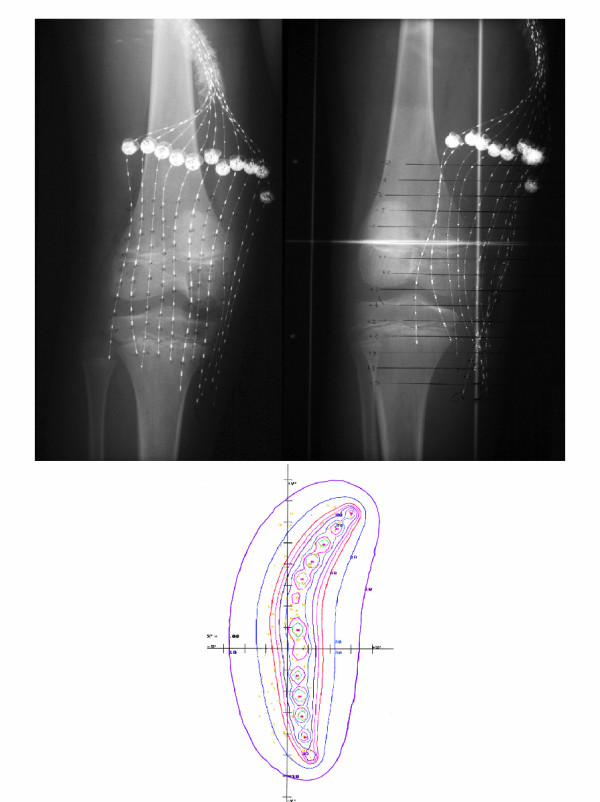
Afterloading catheters and dosimetry (case one).

At the time of his evaluation after referral, he had strong popliteal pulses. There was a 7.0 × 4.5 cm area of swelling and numbness in the left popliteal region. The deep-tendon reflexes were brisk and the motor exam and gait were normal. MR showed residual abnormality consistent with tumor lateral to the joint capsule. Metastatic work-up including nuclear bone scan and CT scan of the chest was negative. Tumor bed re-excision with placement of afterloading catheters was performed in December 1996. All visible residual abnormality was removed without significant disruption of underlying ligaments and tendons. The walls of the tumor bed were biopsied to map the extent of microscopic residual disease. Microscopic residual disease was anticipated given the site of involvement and the limited ability to operate beyond the extent of the abnormal appearing tissues. Eleven afterloading catheters were placed in a parallel array to cover the tumor bed [Figure 2]. Radio-opaque clips were placed at the site of the biopsies and to demarcate the extent of the tumor bed for brachytherapy planning. The final pathology confirmed the presence of residual tumor in the operative specimen and microscopically involved margins at the central and superomedial aspects of the tumor bed.

**Figure 3:**
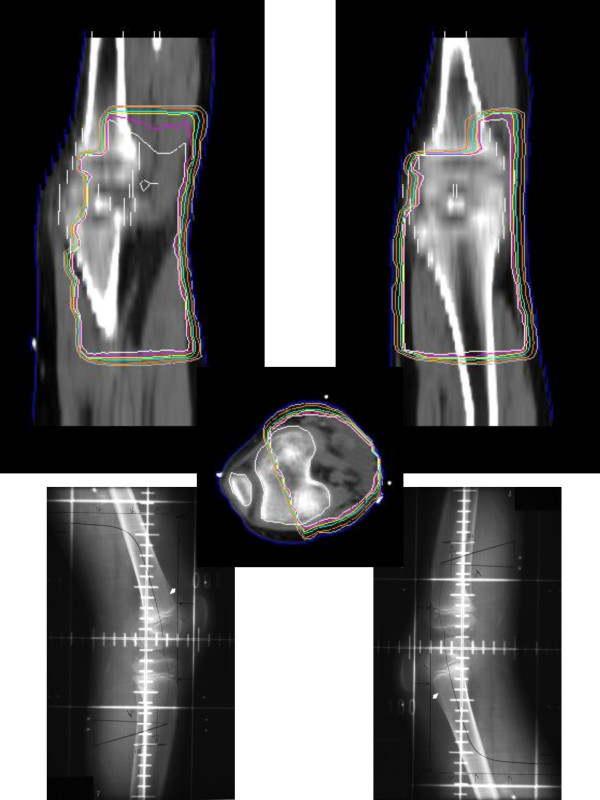
Treatment ports and dosimetry (3D).

Five days after surgery, the 11 catheters were loaded with a total of 135 seeds representing 408 millicuries of I^125^. The dwell time of the implant was 64 hours and the patient received a total implant dose of 2560 cGy delivered at 40 cGy/hr. Three weeks later, the patient began external beam irradiation at 180 cGy per day and received a total external beam dose of 4860 cGy using 6 MV photons with treatment delivered in a parallel-opposed beam arrangement using a CT based treatment plan [Figure 3]. Radiation therapy was completed in February 1997.

A decision was made to initiate chemotherapy three weeks into the course of external beam irradiation based on the perceived high-risk nature of his case – longstanding history of symptoms, known residual tumor and size of tumor at presentation. Chemotherapy included vincristine, ifosfamide, and adriamycin was eventually administered for a total of four cycles. There was central dehiscence of the wound prior to the completion of radiation therapy. The wound was colonized with Enterococci sensitive to ampicillin and managed with antibiotics, whirlpool treatment and daily dressing changes. There was a one-week treatment break during the external beam portion of the treatment.

At the completion of chemotherapy (June 1997) the patient underwent excision of scar tissue with rotation flap of gastrocnemius and skin and Z-plasty of the semi-membranous and semi-tendinous tendon for a non-healing ulcer in the operative region. There was also contracture of the knee joint without signs of abscess or cellulitis. One month later, a second procedure was required to debride and irrigate the left popliteal fossa wound at the site of previously irradiated tissue and contracture release with muscle and fasciocutaneous flap closure. One year after the completion of all therapy (June 1998) the patient reported full range of motion and softening of previously fibrotic tissue. He was actively playing baseball and had no imposed limitations.

**Figure 4:**
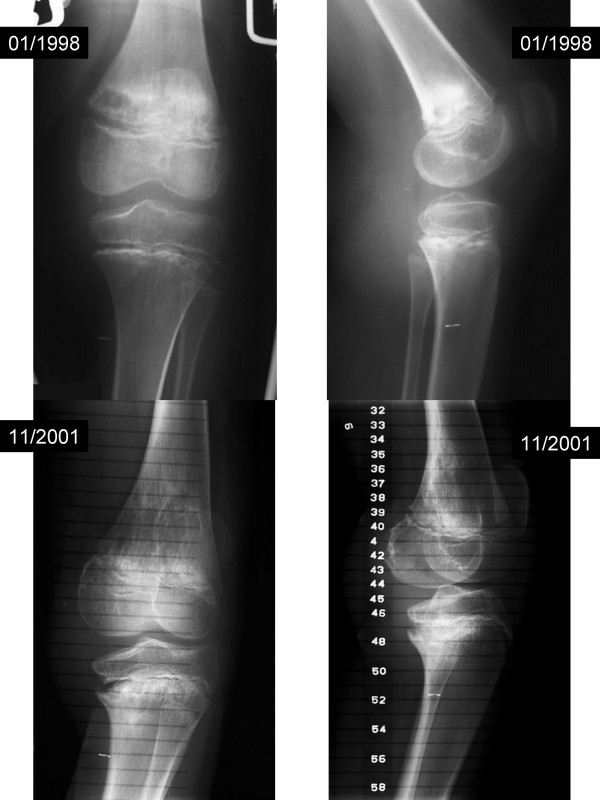
Serial plain films (case one).

**Figure 5:**
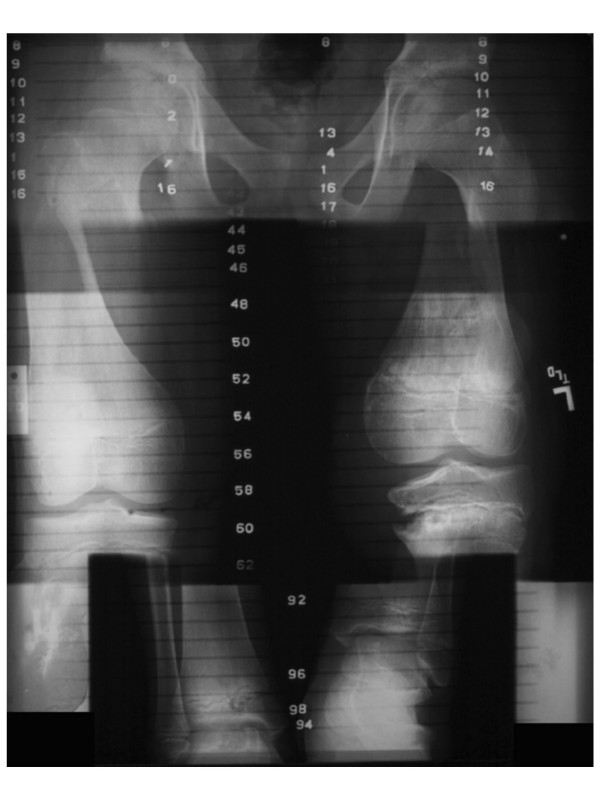
Scanogram 4/2001 (case one).

**Figure 6:**
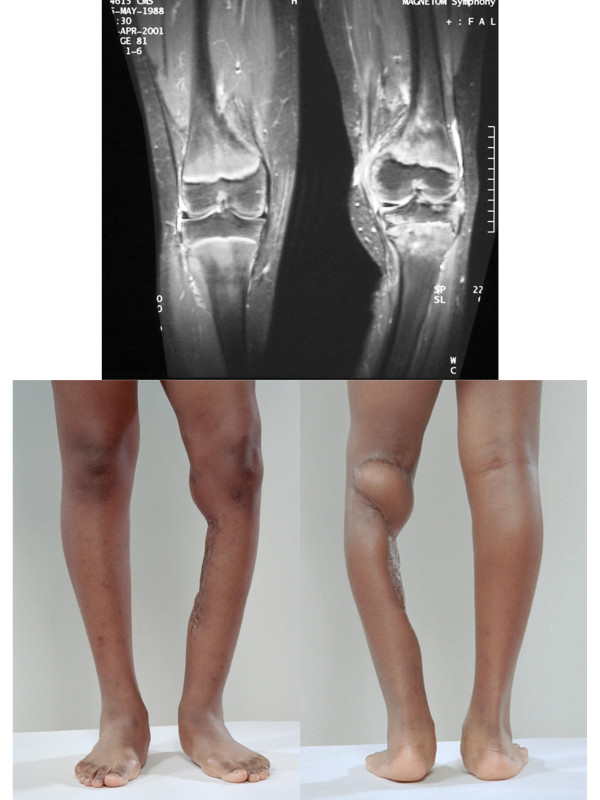
Pre-Op MR and photograph (case one).

Nearly two years after completion of treatment (March 1999), the patient was noted to have Trendelenburg gait after prolonged walking ascribed to poor endurance of weakened hip abductors bilaterally. Hip hiking was also noted on the contralateral side due to leg length and ASIS height discrepancy. A difference of 2.5 cm was noted when measured from the umbilicus to the medial malleolus. He had grown 7.0 cm since the time of diagnosis. He was instructed to stretch the heel cord and strengthening his weak hip abductors. Arrangements were made to provide a shoe lift to accommodate the leg length difference. The discrepancy improved to less than 1 cm over a three month period of time (March-June 1999). He was carefully monitored for growth discrepancy and 6 months later, nearly 3 years after the initiation of treatment, the discrepancy returned to its original value of greater than 2 cm. He also had difficulty with ambulation with slight left-sided limp. Orthopedic surgery was re-consulted. The discrepancy was followed and treated with additional shoe lift. The patient continued to engage in normal activities including sports and reported full range of motion and normal strength. More than four and a half years after initiating treatment (August 2001) [Figure 4], x-ray scanogram revealed estimated lengths of 49.0 cm and 46.5 cm for his right and left femora respectively, and 40.7 cm and 37.0 cm for his right and left tibiae respectively [Figure 5]. There was a clinically noticeable varus deformity of the left knee. All growth plates were still open radiographically. He was taken to surgery five years after the initiation of definitive therapy (December 2001) [Figure 6] for a panepiphysiodesis of the right leg. Using an image intensifier to locate the distal femoral growth plate, medial and lateral incisions were made and a wire-guided cannulated reamer was used to obliterate the plate. The same procedure was done to the proximal tibial growth plate. There were no complications from surgery and the wounds healed appropriately. He was started on physical therapy including quad sets to maintain adequate range of motion. There has been no evidence of tumor recurrence or metastatic disease nearly seven years after treatment. There are no limitations regarding activities and there has been no progressive angular growth deformities.

### Case 2

**Figure 7:**
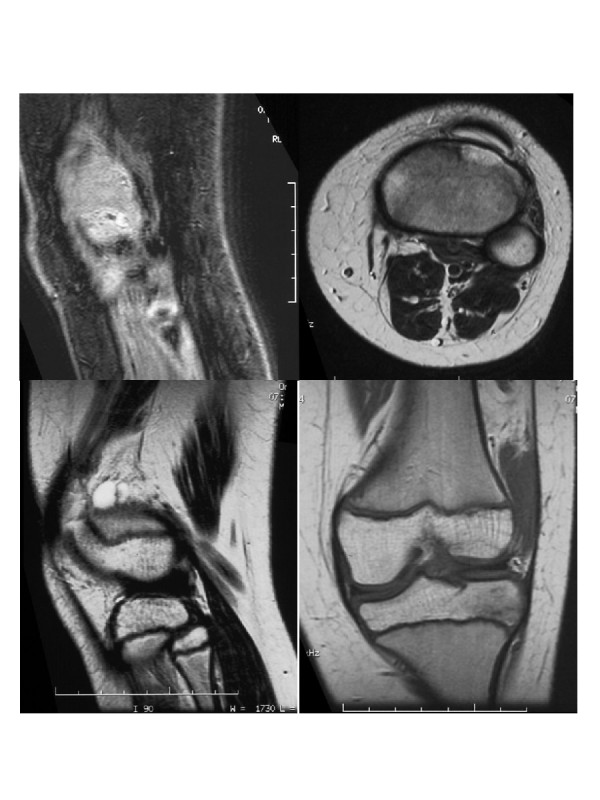
MR Imaging (case two).

At the time of diagnosis this patient was a 9 year-old female with a one year history of pain and swelling about her left knee. She had experienced a fall and related all symptoms to the fall. She was seen in her local emergency room by her family physician; there was no diagnosis or treatment. Approximately one month prior to her representation, she was struck in the left knee by a basketball and developed worsening pain. She was seen by an orthopedic surgeon (December 1999) and was noted to have a valgus posture of both lower extremities, exaggerated on the left by external rotation and she walked with a mild limp. The left knee had no effusion but was hypersensitive to light touch over the lateral aspect where there was soft tissue swelling just below the knee. There was no obvious mass in the area, although firm palpation was difficult because of patient discomfort. Plain films were normal and an MR was ordered that revealed an apparent meniscal cyst in the lateral aspect of the left knee [Figure 7]. Biopsy of the cystic structure was performed (November 1999) that revealed a high-grade synovial cell sarcoma. Metastatic work-up consisting of nuclear bone scan and CT of the chest were negative. Amputation was offered by the local care team that included a radiation oncologist because of their concern about possible contamination of the joint space and uncertain functional outcome. The patient was referred to St. Jude Children's Hospital for further evaluation and treatment.

**Figure 8:**
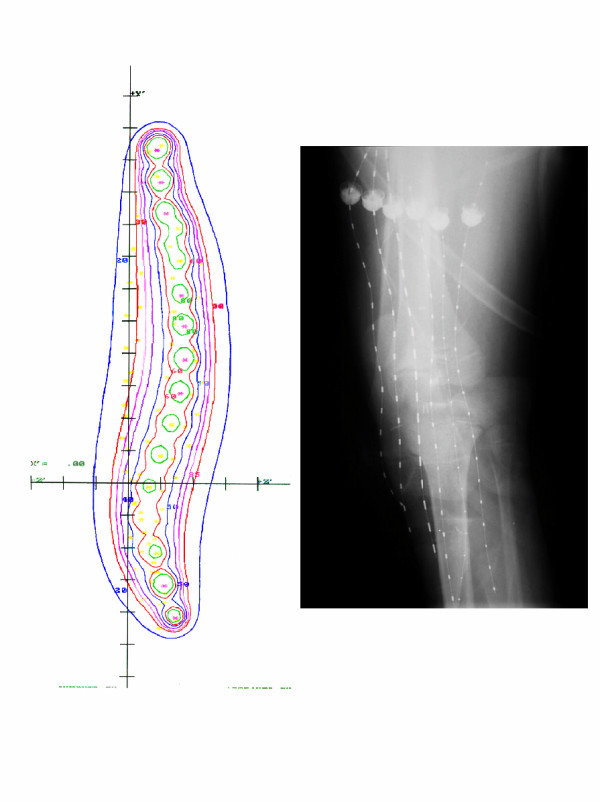
Brachytherapy films and dosimetry.

**Figure 9:**
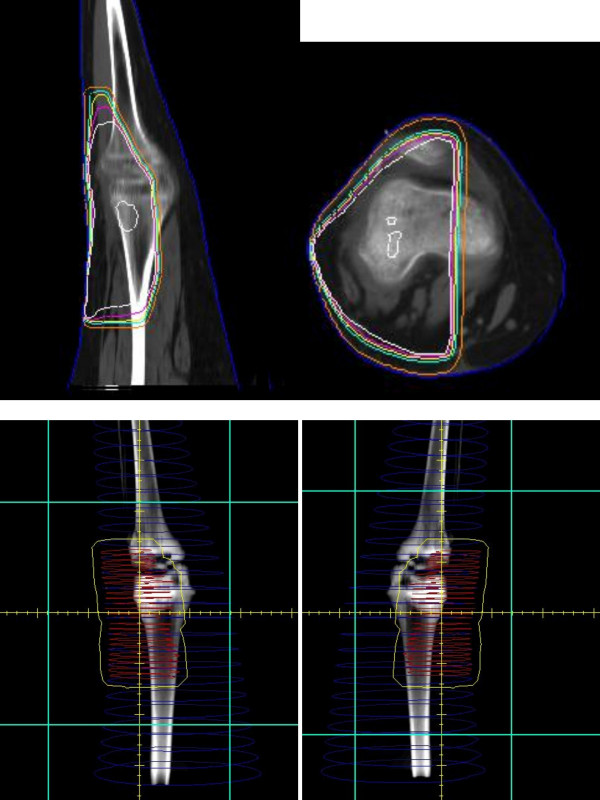
External Beam films and dosimetry.

At the time of her evaluation after referral (January 2000), there was a well healed scar with no excessive swelling. There was mild tenderness on the lateral aspect of her left knee. Additional imaging studies showed abnormality at the site of prior surgery equivocal for residual tumor. There was no evidence of abnormality in the joint space. The tumor bed was explored. There was no physical evidence of compromise at the level of the joint space. She underwent wide local excision with placement of afterloading catheters. Six catheters were placed in a parallel array with 1 cm spacing. Radio-opaque clips were placed to delineate the tumor bed and assist in brachytherapy planning [Figure 8]. The margins of the resection were involved with tumor, as demonstrated by field biopsies and assessment of the margins of resection. Satellite tumor nodules were present in the resection specimen. Four days after surgery the six afterloading catheters were loaded with 82 seeds representing 302 mCi of I^125 ^(Figure). The dwell time of the implant was 62 hours and the patient received a total implant dose of 2480 cGy delivered at 40 cGy/hr. Two weeks later the patient began external beam irradiation at 180 cGy/day and received a course of treatment and total external beam dose of 5040 cGy using 6 MV photons with treatment delivered with two beams using a CT based treatment plan [Figure 9]. Radiation therapy was completed in March 2000.

**Figure 10:**
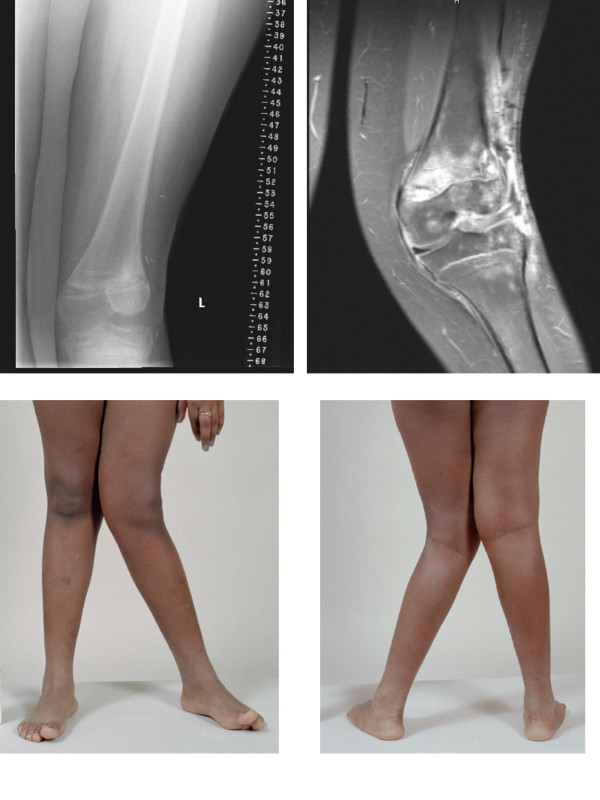
MR Imaging and photography.

The patient suffered moist desquamation corresponding to the radiation therapy portal that was predicted based on the treatment and the use of a tissue equivalent bolus material which was placed on the wound on alternating days during her course of external beam irradiation. She was able to return home on the last day of treatment. On routine follow-up, only 4 months after treatment, left leg appearing to be slightly longer than her right leg by less than 1 cm. No corresponding gait problems were reported. Nearly one year after treatment (January 2000) physical examination showed good range of motion at the left knee; however, there was significant valgus angulation. An MR study was reviewed by Orthopedic Surgery and was noted to show growth arrest laterally and predominantly involving the distal femoral physis [Figure 10]. Based on these findings, the family was informed that an epiphysiodesis of the distal femoral physis would likely be required to prevent additional deformity. Due to the angulatory deformity, an osteotomy of the distal femur would be required. Because of high-dose irradiation and concerns about bone healing, osteotomy and epiphysiodesis were deferred until the three year evaluation was performed. At that time, the patient had a significant valgus deformity. The morbidity of the deformity was such that ambulation was difficult. The patient underwent a closing wedge correcting osteotomy, which was fixed with a contour plate. The patient subsequently fractured the plate secondary to early and unprotected weightbearing (against medical advice). She was placed in a cast and ultimately healed her osteotomy. She continues to have a significant limb length discrepancy and will require future lengthening procedures. She remains without evidence of disease nearly 4 years after treatment.

## Conclusion

Due to its high propensity for local recurrence and metastasis, aggressive treatment of synovial sarcoma is imperative. While many different methods have been used in the treatment of these high-grade tumors, including mono-bloc soft part resection and amputation, the current standard includes local excision and radiation therapy when feasible [16]. Wide local excision with adjuvant radiation therapy is known to achieve a satisfactory rate of local control and good functional outcome [1-15]. Because these tumors commonly arise near tendon sheaths or joint capsules, treatment plans intending to achieve limb conservation may injury the epiphyseal growth plate affecting normal growth and development. Efforts should continue to improve our ability to delineate the tumor, achieve resection with microscopically negative margins and irradiate the region at risk in a manner that minimizes the effect on normal tissues [17].

Both patients in our study underwent two surgical procedures and were treated with brachytherapy and external beam irradiation. Brachytherapy was used to confine the highest doses to the region at risk and minimize the dose received by normal tissues. The use of brachytherapy shortens the overall treatment time and increases the rate of local control in the setting of involved margins of resection. CT-based treatment planning was used to define the volume of irradiation and to spare normal tissue structures. By reducing the amount of radiation dose delivered to normal tissues, the probability of growth deformity, radio-chemotherapy interactions, and even the hypothetical risk of second tumor formation may be lowered. No effort was made to symmetrically irradiate the physes, which would hypothetically lead to symmetrically diminished growth without the added effect of angular deformity. Because of concerns about the effects of total joint irradiation and its possible effects on functional outcome, the inhomogeneous and asymmetric approach was taken. Despite the valgus and varus deformity experienced by these children and the need for intervention, both children and parents were completely satisfied with their functional outcome and indicated that they would chose the same course of treatment if presented again with the same options.

### The rationale for radiation therapy

The importance of achieving local control with aggressive surgery and high-dose irradiation cannot be overemphasized. Local control is crucial to long-term survival and avoiding the morbidity of local tumor progression. Local control, even in the setting of metastatic disease, is an important endpoint. Radiation therapy is highly successful in achieving local control in soft tissue sarcoma and is standard in the care of children with high-grade tumors such as those reviewed in this report. At our institution high-grade tumors with resection margins of 1 cm has an observed local control rate of 72% (5 of 7 patients) in the absence of radiation therapy and 100% (7 of 7 patients) when radiation therapy was given postoperatively. Among 20 unirradiated high-grade tumors that were completely resected with margins > 1 cm only 15 (75%) were locally controlled for an extended period [18]. Our policy is to use external beam irradiation or brachytherapy alone for high-grade tumors that are completely excised, regardless of age or other considerations including anatomic location. We also recommend brachytherapy combined with external-beam irradiation for high-grade tumors with involved, close or indeterminate margins, regardless of size or anatomic location [19]. Low-grade tumors are treated with external-beam radiation therapy or brachytherapy only when the risk of recurrence and re-resection morbidity is high, or at the time of recurrence. These policies apply even to patients with metastatic disease who are likely to survive for an extended period of time after aggressive multimodality therapy including metastasectomy [19,20]. Exceptions may be considered for small, superficial tumors in very young patients when resection can be performed with adequate margins, generally < 5 mm although prospective studies demonstrating the appropriateness of this approach are limited [21].

### Bone growth and development and the effects of various conditions and treatments

Despite efforts to achieve local control and minimize the effects of treatment on normal tissues, damage to bone and soft tissues may be unavoidable. Synovial cell sarcoma commonly arises near tendon sheaths and joint capsules of adolescents and young adults and may be in close proximity to an epiphyseal growth plate during a time of rapid growth. The situation is made worse if the tumor is located around the distal femur or proximal tibia. Among the four epiphyseal plates in the lower extremity that contribute to the growth of the limb, those around the knee make the most significant contribution, with the distal femur and proximal tibia accounting for 50–90% and 57% of limb growth, respectively depending on age [22]. In our study, Case 1 was eight years old at the time of diagnosis and had a tumor lateral to the joint capsule of the left knee. Case 2 was nine years old at the time of diagnosis and had a tumor lateral to the left distal femur and in close proximity to the joint space. It was less than one year after diagnosis and definitive management that both patients developed a clinically significant angular deformity and leg length discrepancy.

There are two non-congenital mechanisms that are known to interfere with growth of the physis: direct trauma and environmental change around the plate. Trauma includes acute injury to the growth plate in manner that affects all or partial growth and results in premature closure or the formation of a physeal bar. Even if the region has retained its ability to grow it is hampered by solid bone formation across the plate [23]. Environmental change is less common and poorly understood. Roberts [24] discussed the disturbance of epiphyseal growth in the knee of infants with osteomyelitis and suggested that damage to the epiphysis might be due to an abscess or ischemia following occlusion of the blood supply. Infection is known to produce more severe leg length discrepancy problems than trauma, because the patients are typically younger at onset [23]. Tumors can contribute to leg length discrepancy either by direct invasion or by originating from the cartilage cells of the physis, thereby stealing growth potential from the plate [25]. Vascular malformations adjacent to the physis have been known to both inhibit and stimulate growth [26,27].

Paralysis is also known to cause of growth inhibition, although the mechanism is poorly understood. Proposed contributors include reduced muscle activity, which indirectly alters the blood supply, and abnormal vasomotor control [23]. Avascular necrosis of the epiphysis can involve the growth plate, which obtains its blood supply from epiphyseal circulation, causing growth inhibition. Peterson described a case in which premature closure of the distal tibial physis occurred in an infant after a temporary but significant episode of vascular insufficiency during surgery to correct developmental dislocation of the right hip [28].

Rogalski et al. [27] observed that the proximity of vascular abnormalities to the epiphyseal growth plate was associated with growth disturbance. In his series, 11 out of 41 patients with extremity angiodysplastic lesions developed either hypertrophy or leg length discrepancy. Although vascular malformations have been associated with both undergrowth and overgrowth, all of the patients in this study with leg length discrepancies had overgrowth of the involved limb. These same authors postulated that increased oxygen uptake and increased flow often associated with such vascular malformation contributed to the alteration in growth.

It is also conceivable that repeated surgeries to the lateral aspect of the knee in both patients played a contributive role. A change in the environment surrounding the physis, such as muscle atrophy following prolonged bed rest, paralysis, or limb-sparing surgery with muscle loss, is known to cause a significant slowing of growth [23]. Although efforts were made to keep scarring from surgery to a minimum, re-resection of tumor combined with the damaging effects of radiation to soft tissues would inevitably cause a decrease in tissue vascularity potentially leading to subclinical or clinical necrosis, causing a continual mechanical compression of the physis and retarding growth. Such damage has been documented in heat-related injuries to extremities in which circumferential eschar causes a prolonged ischemia to the physis and subsequent growth inhibition [29].

The patients included in this report had many of the above noted contributions to abnormal growth and development including a pre-existing condition, vascular compromise due to multiple surgeries, loss of muscle mass, limited use of the extremity for a defined period of time, infection and chemotherapy. Chemotherapy may temporarily reduce bone growth through systemic effects that include the direct effects of specific agents or the indirect effects resulting from systemic infections and abnormalities in metabolism and nutrition.

### The effects of irradiation on bone growth

It has been known for almost a century that radiation therapy at sufficient levels can affect growing bone. Several factors contributing to the severity of effect including the total dose, dose per fraction, dosimetry (asymmetry and inhomogeneity) and age at the time of irradiation [24,30,31]. Probert and Parker [32] studied the standing and sitting height of 44 children who underwent total spinal irradiation for Hodgkin's disease, medulloblastoma or acute lymphoblastic leukemia. Among the patients receiving more than 3,500 rads of spinal irradiation, 8 out of 29 (28%) had a sitting height more than 2 standard deviations below the mean for age. Among those receiving less than 2,500 rads, 6 out of 15 (40%) had a sitting height more than 2 standard deviations below the mean for age. They concluded that doses in excess of 2,000 rads affect vertebral body growth in children. They further noted that children less than six years of age or those undergoing puberty experienced the most significant damage, suggesting that there is an increased sensitivity of bone to irradiation during specific developmental periods.

The conclusions of Gonzalez and Breur [33] were slightly different. In their study, that included 22 patients who experienced growth retardation of long bones as the result of radiotherapy in childhood, definitive limb shortening was strongly dependent on the age of the patient when the irradiation treatment began. When the growth remaining after irradiation was taken into account, no differences in radioresponsiveness were apparent. Their results suggested that despite, a temporary decrease in growth rate, irradiated bone will eventually grow at a similar rate to unirradiated bone. The total dose administered had a major influence on limb shortening as higher doses produced a greater effect. The authors noted that a "saturation dose" was apparent at 40 Gy because higher doses did not appear to produce further considerable increase in shortening.

The epiphyseal growth plate is the area of the developing skeleton most sensitive to the effect of radiation due in part to its rapidly proliferating stem cell population. Even low doses of radiation have been shown to cause histologic changes including temporary swelling, fragmentation, and degeneration of chondrocytes [31]. When higher doses are given, permanent changes including necrosis and premature closure of the physis become evident. Such was the case in our study, as both patients received a total radiation dose of over 50 Gy regionally and nearly 75 Gy focally, which undoubtedly contributed to the observed leg length discrepancies. Furthermore, the angular deformity can be attributed to the unequal dose distribution across the physis as depicted in Figures 3 and 9.

### Orthopedic intervention for valgus deformity

Creating a treatment plan for patients with leg deformities who have undergone radiation therapy with or without chemotherapy can pose a difficult challenge. Because the osteocytes of neighboring bone are also destroyed, it may take years for the bone to revascularize and repopulate with healthy osteocytes [23]. The absence of healthy osteoblasts and precursors make lengthening procedures difficult due to unpredictable healing. Radiation damage to regional soft tissues is also an important consideration when planning a lengthening procedure. For these reasons, we decided to delay intervention to correct both the angular deformity and leg length discrepancy, and try to minimize further progression of leg deformity. At the time of surgery, Case 1 had a leg length discrepancy measuring 6 cm clinically. It was felt that epiphysiodesis of the growth plates in the healthy right knee should be the initial treatment, as it would halt progression of discrepancy and allow for some degree of correction, albeit unpredictable because of radiation damage to the growth plates of the left knee. Because of significant angular deformity, case 2 required an osteotomy to correct the defect. Future procedures are planned to address the anticipated leg length inequalities.

Historically, there have been two primary treatments for patients with angular limb deformities: epiphysiodesis and stapling. Both methods seek to achieve the same result while offering different sets of advantages and disadvantages. Partial epiphysiodesis of the knee to correct angular deformity was first described by Phemister in 1933 [34]. It has been used for the correction of idiopathic genu valgum or varum in the adolescent patient. Bowen [35] described a common surgical technique in which a bone block, centered on the physeal line, was removed through a 2 cm incision, rotated 90 degrees, and reset. This procedure causes growth arrest on the treated side and allows for continued growth and self-correction on the opposite side. Advantages to partial epiphysiodesis include a good assessment of further growth using the Green and Anderson technique [36], small surgical scar and high predictability of self-correction due to permanent physeal ablation. Disadvantages involve its confinement for use in adolescents due to the irreversibility of the physeal ablation. Also, estimation of skeletal maturity is difficult and unreliable. Physeal stapling, an alternative method, was first reported by Blount and Clark in 1949[37]. They used stainless steel staples to produce reversible growth retardation and it remains the only reversible means of manipulating growth. This procedure has traditionally been used for adolescents [38].

Newer radiation delivery techniques including high-dose rate brachytherapy, intraoperative radiation therapy [39,40] and the spectrum of conformal external beam radiation therapy planning and delivery techniques [17] seek to confine the prescription dose to the region at risk and minimize the dose received by normal tissues. Computerized treatment planning technology and the use of 3-dimensional imaging permits the delineation of both target and normal tissue structures to the extent that the dosimetry for a defined normal tissue structure, such as bone or soft tissue, may be known with a high degree of precision. This information can be used relatively to compare different treatment plans for a given patient. Prospectively assessed, this information may be used as a clinical variable to correlate treatment dosimetry to abnormalities in growth and development including their time to onset and severity [41]. Until more complete knowledge is available regarding the effects of 3-dimensional dosimetry on bone and soft tissue, the full benefit of these newer treatment techniques will not be realized. We are concerned about the effects of newer treatment technology and the use of more focal irradiation. More focal treatment is likely to result in inhomogeneity and asymmetric irradiation of growth elements in bone. Prospective assessment of the use of these techniques is required.

Limited surgery and irradiation may result in growth abnormalities and deformity. These effects may have minimal or significant impact depending on functional outcome and the value attached to limb preservation for a particular patient. As mentioned in this report, both patients and families were queried about their decisions regarding treatment and both reported satisfaction with outcome recognizing that side effects were anticipated. Both families attached a high value to limb preservation.

## Competing Interests

None declared.

## Authors' Contributions

DTF reviewed the patient records and drafted the manuscript. WCW contributed to drafting the manuscript. MDN contributed to drafting the manuscript. TEM conceived of the study, participated in the review of the data, and helped draft the manuscript. All authors read and approved the final manuscript

## Pre-publication history

The pre-publication history for this paper can be accessed here:


